# Commentary: Should we worry about climate worry? Understanding and promoting youth resilience and pro‐environmental engagement: a commentary on Park et al. (2026)

**DOI:** 10.1111/camh.70091

**Published:** 2026-03-25

**Authors:** Stathis Grapsas, Atheer Massarwe

**Affiliations:** ^1^ Utrecht University Utrecht The Netherlands; ^2^ The Hebrew University of Jerusalem Jerusalem Israel

**Keywords:** Adolescence, climate emotions, climate worry, mental health, pro‐environmental behavior

## Abstract

Understanding the mental health impact of climate change is of paramount importance to promoting youth resilience and pro‐environmental engagement. Here, we reflect on the study of Park et al. (*Child and Adolescent Mental Health*, 2026) on the prevalence and mental health correlates of climate worry in young adolescents. We propose that terminological clarity and abstinence from evaluative judgments on the adaptiveness of climate worry are necessary to unravel its role and function in young people's mental health and lives. We also provide suggestions for future research and practice that aim to deepen on three key implications of the study: (a) that climate worry has multiple unique links to a host of mental health problems in young adolescents; (b) that gender differences in climate worry are present already from a young age; and (c) that adults should promote both resilience and pro‐environmental engagement in youth, in a developmentally appropriate manner.

The youth of today are growing up in a world of climate emergency. They are the first generation to experience from an early age the destructive consequences of global warming. Compared to older generations, they are disproportionately impacted by it. While navigating a life period in which they still develop the skills to regulate and cope with negative emotions, climate change is progressing at alarming levels and global inaction undermines its resolution—a warning that the worst climate change consequences are yet to be felt. Understanding the mental health impact of climate change is therefore an imperative toward shielding mental health while simultaneously promoting pro‐environmental engagement for the youth of today and of the future.

Research that centers on this subject is in its early days, and especially scarce when it comes to early adolescents. The article of Park, McArthur, Tough, and Madigan (2026) entitled “Prevalence, conceptual distinctiveness, and cross‐sectional correlates of climate worry in Canadian adolescents” is a promising endeavor in this direction. Using a large, gender‐balanced sample of early adolescents from the Canadian Alberta province (a region facing floods and wildfires while maintaining strong ties to the fossil fuel industry), the article provides findings on the prevalence and correlates of climate worry, and a discussion that addresses important issues for future research and practice. In the present commentary, we embed the contributions and limitations of this article in a broader discussion of key topics for theory, research, and practice in the domain of youth mental health and environmental engagement.

## Climate worry as the cognitive aspect of climate anxiety

Climate worry is a lingering concern that climate change will have dire consequences if not resolved. Climate worry contains an affective and a cognitive component and is in essence an affectively motivated cognition. What Park et al. (2026) did not point out is that the affective component of climate worry is essentially climate anxiety (and vice versa, the cognitive component of climate anxiety is climate worry – cf. Stewart, Chapman, & Davis, 2023). Attesting to this, across a series of studies with adolescents (Becht et al., 2024), *feelings* of climate worry and anxiety were highly interrelated, and the authors referred to their composite as climate anxiety affect. In these studies, climate anxiety affect was found moderately related to clinical symptoms of climate anxiety and modestly to moderately related to symptoms of generalized anxiety and pro‐environmental behavior, a pattern similar to Park et al. (2026). Research on the structure of climate emotions in adults also resonates with this perspective (Marczak et al., 2023). Thus, insights from research on climate worry extend to research on climate anxiety and vice versa, allowing a better synthesis of the two literatures. Synthesizing such findings at a conceptual level can help the emerging theory and research on the structure, dynamics, and correlates of climate emotions move forward with more theoretical precision and terminological parsimony.

## Moving from evaluative to functional questions

Setting this conceptual issue aside, there are important takeaways from the study. An important and novel finding is that young adolescent climate worriers tend to experience a host of mental health struggles that extend beyond the field of environmental issues, above and beyond their preexisting generalized anxiety symptoms: lower optimism and happiness, as well as elevated depression, social media and video game addictions, and sleep disturbances. Should we worry about climate worriers? We believe yes, both for scientific and for youth mental health reasons. Environmental psychology researchers often frame climate worry and anxiety as practical and adaptive because they are justified responses to climate change and because they promote pro‐environmental behavior. From a mental health perspective that considers the present findings and the authors' discussion thereof, such framing appears incomplete: first, because it focuses on an outcome of different *quality* to assess adaptiveness (pro‐environmental behavior, not broader aspects of biopsychosocial functioning), and second because it assesses the adaptiveness of climate worry solely on the grounds of how it *helps resolve the triggering problem* (via increased pro‐environmental behavior) but not on the grounds of how it may *affect broader psychological development and functioning*, either positively (e.g., via upholding personal morals and giving purpose in life) or negatively (e.g., via psychological disturbances that settle in).

Acknowledging the warning signs of the current findings is a key issue for a more integrative theory on mental health vis‐à‐vis climate change. Instead of asking *evaluative* questions (e.g., whether climate worry is adaptive or maladaptive), we can focus on *functional* questions that help us understand *when, why, how, for whom*, and *toward what ends* climate worry is beneficial or obstructive. Experimental, longitudinal, daily diary, and interview studies can shed light on the multifaceted causes, daily dynamics, developmental trajectories, consequences, and meanings of climate emotions for youth from diverse cultural and demographic backgrounds, with varying forms and degrees of lived climate change impact and environmental (dis)engagement. Research that informs these questions is especially important in early adolescence. This is a sensitive developmental period that involves rapid biopsychosocial transitions, and in which emergent mental health struggles can offset developmental trajectories of long‐term mental health problems. Research of this sort can both borrow from and contribute to the field of mental health. This latter field is increasingly finding a balanced footing that considers the dynamic interplays of person, context, and time in human psychology: a shift from black‐and‐white perspectives on what negatively valenced emotions are and how they should be regulated to nuanced accounts of the conditional benefits and drawbacks of experiencing them and up‐ or downregulating them. Such dynamics are likely relevant to climate emotions.

## Understanding gender differences

Another important finding concerns gender identity differences in climate worry. Like other studies in adolescence and adulthood, Park et al. (2026) found that male youth are, on average, less worried about the climate than female and gender‐minority youth. This recurring finding makes sense: environmentalism is associated with empathy and communal values, and the tendency to experience more frequently and intensely negative emotions such as worry is a hallmark of neuroticism, characteristics that are on average less present in males. It is compelling to gloss over this gender difference, yet the fact that it is present in early adolescence warrants further examination because the findings concern large groups of young people found across countries and cultures.

The origins and dynamics of these gender differences may be traceable across different levels. At the interpersonal level, it is possible that the discussion around climate change becomes gendered from an early age, affecting how youth learn about, engage with, and are psychologically impacted by environmental issues in their daily life. This may be also happening inadvertently and indirectly, when adults use language and appeal to values that young males may on average find less motivating. At the intrapsychic level, gender differences in cognitive style, emotion processing, and emotion regulation may contribute to differences in the triggers and intensity of climate worry, as well as in the degree to which climate worry impacts mental health and motivates pro‐environmental engagement. We thus need to understand better *why* some prototypically gendered attributes are more decoupled from or coupled with concern about environmental issues, and *how* psychological processes, daily experiences, socialization, and climate education encourage or discourage pro‐environmental engagement and psychological well‐being in the face of climate change. Doing so may not only identify facilitators and barriers of mental health and pro‐environmental (dis)engagement that we have potentially overlooked but also aid in understanding the intersectionality of multiple identities that young adolescents are in the process of forming (e.g., gender identity, political identity, pro‐ or antienvironmental identity), as well as its consequences. Research on these topics can eventually provide insights into how to promote pro‐environmental engagement and resilience in the face of climate change among those young people who seem to need it the most.

## Promoting youth resilience and pro‐environmental engagement in the face of climate change

Taken together, the study findings call for a two‐pronged action: promote mental health and pro‐environmental engagement in a synergistic, age‐appropriate manner. As Park et al. (2026) insightfully note, adults need to be prepared to address adolescents' worries surrounding climate change. Not only by dismissing them or attempting to dissolve them but also by validating them and using them as catalysts of resilience, environmental efficacy, and pro‐environmental engagement. Equally importantly, adults should also instill resilience and pro‐environmental engagement in the less climate‐engaged and climate‐worried youth. These tasks are especially challenging in adolescence. Alongside well‐known findings on mental health dips, some research suggests that pro‐environmental behavior also dips on average during this period (e.g., Otto, Evans, Moon, & Kaiser, 2019). Accordingly, interventions to promote pro‐environmental engagement in adolescence are often less successful.

Why may this be? Traditional, prescriptive approaches to promote pro‐environmental engagement for the sake of a better future by adult authority figures like parents and teachers often do not land well and are met with resistance, because they misalign with the core features of adolescent psychology: adolescents tend to focus on the “here and now,” and are especially driven to gain agency in their personal lives, autonomy from parents, and acknowledgment (or status) from their peers. While these psychological features often lead to ill‐advised risks (e.g., substance abuse), they can also lead to productive risks, such as those associated with pro‐environmental activism (e.g., Fridays for Future movement). Inspired by their potential to offset pro‐environmental engagement, our research group studies how to promote pro‐environmental behavior in ways that synergize with these features of adolescent psychology. We call this approach “motive‐alignment” (Thomaes, Grapsas, van de Wetering, Spitzer, & Poorthuis, 2023). Instead of framing pro‐environmental behavior as a prescriptive necessity (a “should”) to prevent distal disasters, in the experimental interventions that we develop together with adolescents, we frame it as a collective “can”: as behavior that is *already a norm* in some youth groups (aligned with adolescents' motive for peer status), and that allows younger generations to *make a positive change in society* here and now by striving for fairness and environmental justice (aligned with adolescents' motives for agency and autonomy). Through interviews and focus groups, adolescents guide us in interpreting findings of our prior research, in creating educational materials with a perspective, argumentation and language that speaks to their peers, and in selecting stimuli that will deliver the message more effectively (e.g., Spitzer et al., in press; van de Wetering, Grapsas, Poorthuis, & Thomaes, 2025).

The motive‐alignment approach has shown, across cultures, incremental success in raising adolescents' environmental efficacy, and pro‐environmental behavior overtraditional climate education approaches that attempt to do so by prescribing what adolescents “should” do (Spitzer et al., in press; van de Wetering et al., 2025). Importantly, these findings underscore that adolescents can be especially motivated by adults when they feel *empowered* and *inspired*. Pro‐environmental initiatives to further address climate worry and raise youth resilience in the face of climate change can build on these insights. A major challenge in setting up and carrying out these initiatives is how much scientists, professionals, and parents themselves are ready to act in a manner that speaks to adolescents. To level, listen and co‐create with them; to balance guidance and autonomy; and to trust that adolescents can acquire the tools to cater for their mental health, create their vision for a sustainable society and initiate a plan of action to make it a reality.

## Conflict of interest statement

The authors have declared that they have no competing or potential conflicts of interest.

## Funding information

This work was supported by the European Research Council (ERC) GREENTEENS Project (Consolidator grant no. 864137) and BeValue Project (Consolidator grant no. 101087000).

## Ethical information

Obtaining consent or institutional approval by the Ethics Review Board was not necessary, as this work does not include participant data.

## Data Availability

Data sharing is not applicable to this article, as no datasets were generated or analyzed during the current study.

## References

[camh70091-bib-0001] Becht, A. , Spitzer, J. , Grapsas, S. , van de Wetering, J. , Poorthuis, A. , Smeekes, A. , & Thomaes, S. (2024). Feeling anxious and being engaged in a warming world: Climate anxiety and adolescents' pro‐environmental behavior. Journal of Child Psychology and Psychiatry, 65, 1270–1282. 10.1111/jcpp.14035 38940197

[camh70091-bib-0002] Marczak, M. , Wierzba, M. , Zaremba, D. , Kulesza, M. , Szczypiński, J. , Kossowski, B. , … & Marchewka, A. (2023). Beyond climate anxiety: Development and validation of the Inventory of Climate Emotions (ICE): A measure of multiple emotions experienced in relation to climate change. Global Environmental Change, 83, 764.

[camh70091-bib-0003] Newman, M.G. , Llera, S.J. , Erickson, T.M. , Przeworski, A. , & Castonguay, L.G. (2013). Worry and generalized anxiety disorder: A review and theoretical synthesis of evidence on nature, etiology, mechanisms, and treatment. Annual Review of Clinical Psychology, 9, 275–297. 10.1146/annurev-clinpsy-050212-185544 PMC4964851 23537486

[camh70091-bib-0004] Otto, S. , Evans, G.W. , Moon, M.J. , & Kaiser, F.G. (2019). The development of children's environmental attitude and behavior. Global Environmental Change, 58, 947.

[camh70091-bib-0005] Park, J. , McArthur, B.A. , Tough, S. , & Madigan, S. (2026). Prevalence, conceptual distinctiveness, and cross‐sectional correlates of climate worry in Canadian adolescents. Child and Adolescent Mental Health. 10.1111/camh.70076 PMC13668473 41704206

[camh70091-bib-0006] Spitzer, J. , Grapsas, S. , Poorthuis, A.M.G. , Li, F. , Song, Y. , & Thomaes, S. (in press). Motive‐alignment promotes adolescents' pro‐environmental behavior: A field experiment in two cultures. *Psychological Science*. 10.1177/09567976261440239 PMC13424257 42113699

[camh70091-bib-0009] Stewart, A.E. , Chapman, H.E. , & Davis, J.B.L. (2023). Anxiety and Worry about Six Categories of Climate Change Impacts. International Journal of Environmental Research and Public Health, 21, 23. 10.3390/ijerph21010023 PMC10815757 38248488

[camh70091-bib-0007] Thomaes, S. , Grapsas, S. , van de Wetering, J. , Spitzer, J. , & Poorthuis, A. (2023). Green teens: Understanding and promoting adolescents' sustainable engagement. One Earth, 6, 352–361.

[camh70091-bib-0008] van de Wetering, J. , Grapsas, S. , Poorthuis, A. , & Thomaes, S. (2025). Shifting peer norms, shifting behavior? Optimizing educational interventions to promote adolescents' sustainable dietary choices. Journal of Environmental Psychology, 102, 880.

